# Implementation of a novel PCR based method for detecting malaria parasites from naturally infected mosquitoes in Papua New Guinea

**DOI:** 10.1186/1475-2875-8-182

**Published:** 2009-08-01

**Authors:** Arif U Hasan, Setsuo Suguri, Jetsumon Sattabongkot, Chigusa Fujimoto, Masao Amakawa, Masakazu Harada, Hiroshi Ohmae

**Affiliations:** 1 Department of International Medical Zoology, Faculty of Medicine, Kagawa University, 1750–1, Ikenobe, Miki, Kita, Kagawa, 761-0793 Japan; 2 Department of Entomology, U.S. Army Medical Component, Armed Forces Research Institute of Medical Sciences, 315/6 Rachavithi Rd., Bangkok, 10400 Thailand; 3 Department of Medical Technology, Faculty of Health Sciences, Kagawa Prefectural College of Health Sciences, Hara, Mure, Takamatsu, Kagawa, 761-0123 Japan; 4 Department of Parasitology, National Institute of Infectious Diseases, 1-23-1, Toyama, Shinjuku, Tokyo, 162-3640 Japan

## Abstract

**Background:**

Detection of *Plasmodium species *in mosquitoes is important for designing vector control studies. However, most of the PCR-based detection methods show some potential limitations. The objective of this study was to introduce an effective PCR-based method for detecting *Plasmodium vivax *and *Plasmodium falciparum *from the field-caught mosquitoes of Papua New Guinea.

**Methods:**

A method has been developed to concurrently detect mitochondrial cytochrome b (*Cyt b*) of four human *Plasmodium *species using PCR (*Cytb*-PCR). To particularly discriminate *P. falciparum *from *P. vivax*, *Plasmodium ovale *and *Plasmodium malariae*, a polymerase chain reaction-repeated fragment length polymorphism (PCR-RFLP) has further been developed to use with this method. However, due to limited samples number of *P. ovale *and *P. malariae*; this study was mainly confined to *P. vivax *and *P. falciparum*. The efficiency of *Cytb*-PCR was evaluated by comparing it with two 'gold standards' enzyme linked immunosorbent assay specific for circumsporozoite protein (CS-ELISA) using artificially infected mosquitoes; and nested PCR specific for small subunit ribosomal RNA (*SSUrRNA*) using field caught mosquitoes collected from three areas (Kaboibus, Wingei, and Jawia) of the East Sepic Province of Papua New Guinea.

**Results:**

A total of 90 mosquitoes were artificially infected with three strains of *Plasmodium*: *P. vivax-*210 (*n *= 30), *P. vivax*-247 (*n *= 30) and *P. falciparum *(*n *= 30). These infected mosquitoes along with another 32 unfed mosquitoes were first checked for the presence of *Plasmodium *infection by CS-ELISA, and later the same samples were compared with the *Cytb*-PCR. CS-ELISA for *P. vivax*-210, *P. vivax*-247 and *P. falciparum *detected positive infection in 30, 19 and 18 mosquitoes respectively; whereas *Cytb*-PCR detected 27, 16 and 16 infections, respectively. The comparison revealed a close agreement between the two assays (κ = 0.862, 0.842 and 0.894, respectively for Pv-210, Pv-247 and *P. falciparum *groups). It was found that the eight CS-ELISA-positive mosquitoes detected negative by *Cytb*-PCR were false-positive results. The lowest detection limit of this *Cytb*-PCR was 10 sporozoites. A highly concordance result was also found between nested PCR and *Cytb*-PCR using 107 field caught mosquitoes, and both tests concordantly detected *P. falciparum *in an *Anopheles punctulatus *mosquito collected from Kaboibus. Both tests thus suggested an overall sporozoite rate of 0.9% (1/107) in the study areas. Subsequently, PCR-RFLP efficiently discriminated *P. falciparum *from *P. vivax *for all of the *Cytb*-PCR positive samples.

**Conclusion:**

A single step PCR based method has been introduced here that is highly sensitive, efficient and reliable for identifying *P. vivax *and *P. falciparum *from mosquitoes. The reliability of the technique was confirmed by its ability to detect *Plasmodium *as efficiently as those of CS-ELISA and nested PCR. Application of the assay offers the opportunity to detect vector species of Papua New Guinea and may contribute for designing further vector control programmes.

## Background

Identification of the *Plasmodium *species in the mosquito is a prerequisite for understanding the ecology, geographical distribution, abundance and behaviour of the vector species [1]. It is particularly important in geographical areas where malaria is intense and transmitted by a number of mosquito species. One such country is Papua New Guinea. According to the World Malaria Report 2008, in the year 2006 this country had about 1.5 million malaria cases and almost 3,000 deaths [2]. Members of the *Anopheles punctulatus *group, widely distributed in the southwest Pacific, are responsible for transmitting this deadliest disease, malaria, within this region [3-5]. This group contains at least 12 members:* Anopheles farauti*, *Anopheles hinesorum*, *Anopheles torresiensis*, *An. farauti *Nos. 4, 5, 6, *Anopheles irenicus*, *Anopheles punctulatus*, *An*. sp. near *punctulatus*, *Anopheles koliensis*, *Anopheles rennellensis *and *An. clowi *[3-6]. Recently, Bower *et al *[7] reported an additional putative taxon, *An. farauti *8, endemic in some parts of Australia. However, except *An. irenicus *and *An. farauti *8, Papua New Guinea harbours all of the members of the group (see [3] for a review). Among them *An. farauti*, *An. hinesorum*, *An. punctulatus *and *An. koliensis *are abundantly collected by human bait, and generally considered as major vectors of Malaria [8]. Interestingly, on the islands of Papua New Guinea [9], Solomon Islands [10] and Vanuatu [11]* An. farauti *readily attract to feeds on humans, whereas in Australia this species shows more zoophilic than anthropophilic feeding behaviour [12]. Beside this, *An. punctulatus*, which plays a major role in transmitting malaria in Papua New Guinea [13], is anthropophilically inert on the islands ([9,14], see also [10]). Recently, *An. farauti *4 has also been collected by human bait in Papua New Guinea [13,15], and even *Plasmodium *has been detected in this species [13]. This versatile nature of the vectorial efficiencies of members of the *An. punctulatus *group in Papua New Guinea warrants a simple, accurate and standard *Plasmodium *identification method from mosquitoes to enforce effective malaria control programmes.

Direct observation of a parasite under microscope is the most reliable method, but it requires fresh materials, which are often difficult to rear for later studies, even in standard laboratories. Moreover, it requires experienced microscopists for accurate identification yet cannot differentiate species [16]. Fluorescent stained mosquitoes can easily be observed under microscope, but this technique itself limits its utility for field-collected samples as it requires alive mosquitoes to be infected artificially by fluorescent labelled parasites [17]. Monoclonal antibodies against the circumsporozoite (CS) protein has been introduced as an alternative to microscopy [18]. Sensitivity and specificity of CS-ELISA is high, and parasite quantification is also possible by this method. Therefore, CS-ELISA is now widely accepted as a 'gold standard' for *Plasmodium *species identification from mosquitoes. In a controlled laboratory using reared mosquitoes, CS-ELISA is definitely a reliable method, but it may bias the results in field-collected materials. Several studies have reported non-specific amplification, failure to detect immature sporozoites in oocysts and overestimation of true salivary gland infection rates using CS-ELISA ([1,19] and references therein). Besides these factors, CS-ELISA is unsuitable for materials preserved in ethanol and requires each assay to run separately for different *Plasmodium *species [20,21]. A rapid dipstick immuno-chromatographic assay (Vec-Test™ Malaria) also showed rapid and accurate means for detection of different *Plasmodium *species, similar to that of CS-ELISA [22]. However, the PCR has proven to be a more sensitive method for the diagnosis of all four species of human malaria parasites [23] and useful tool than the dipstick assay to determine the malarial infection rate in mosquitoes [24]. Conventionally, small subunit ribosomal RNA (*SSUrRNA*) is PCR amplified for this purpose, and these PCR based methods exceed the sensitivity of microscopic examination with the detection limit between three to ten sporozoites in a mosquito [16,18,19,23,25]. Of the currently available sensitive PCR assays for identifying *Plasmodium *from mosquitoes, each requires salivary gland or mid-gut dissection [19], overnight preservation before DNA extraction [16], southern hybridization [18], multiple or separate species-specific reactions (nested PCR) [23,26]. Yet, some one step tests failed to produce reliable and diagnostic PCR products [1,27]. Nevertheless, a selection of appropriate primers [28], storage method of mosquito samples [1] and foremost extraction methods [29] all can affect PCR performance. Therefore, some proven 'gold standard' methods may give substandard results for detecting *Plasmodium *from mosquitoes [1]. Recently, highly specific RT-PCR has been developed for this purpose, and this technique shows promising results [1].

However, there is always a need for an improved method, which will be rapid, applicable to any types of preserved materials, convenient to implement, reliable and has high sensitivity. To meet these purposes, a novel single step PCR test (*Cytb*-PCR), based on mitochondrial cytochrome b (*Cyt b*) gene has been described here. A species-specific polymorphism has been exploited to specifically discriminate *Plasmodium falciparum *from other three species, *Plasmodium vivax*, *Plasmodium ovale *and *Plasmodium malariae*, for polymerase chain reaction-repeated fragment length polymorphism (PCR-RFLP). The prerequisite for each new technology is its validation against accepted 'gold standard' [25]. The *Cytb*-PCR along with the PCR-RFLP were extensively validated with the conventional 'gold standard' CS-ELISA using artificially infected mosquitoes. A trial of naturally infected field-collected mosquitoes from East Sepic province of Papua New Guinea has also been presented here. For validation, the results of this field trial is also presented together with one of the PCR based 'gold standard' approach, nested PCR reported by Snounou *et al *[30].

## Methods

### Mosquito infection

*Anopheles dirus *mosquitoes, reared at the Armed Forces Research Institute of Medical Science (AFRIMS), Bangkok, Thailand, were artificially infected with *P. vivax *or *P. falciparum *using membrane feeding procedure as described in [31]. Briefly, blood was collected from the patients who were diagnosed to have *P. vivax *or *P. falciparum *by the Giemsa-stained thick and thin blood films with standard microscopy according to WHO recommendations. All volunteers for blood donation were explained about the use of their blood and signed a consent form before their blood was collected. The packed red blood cells were washed twice with RPMI culture medium (without serum) then resuspended in AB naïve serum at 1:1 volume, mixed well and added to the blood feeder. The mosquitoes were allowed to feed for 30 minutes. To examine parasite development, guts of some randomly chosen mosquitoes from each batch fed with *P. vivax *infected blood were dissected and checked for oocysts 7 days after feeding, and subsequently salivary glands for sporozoites 14 days after feeding. Similarly, mosquitoes fed with *P. falciparum *infected blood were checked at 9 and 16 days for guts and glands respectively for the same purpose. Sporozoite rate was determined for both species by counting the number of sporozoites in the dissected salivary glands. To explicit the strength of the new method, each mosquito samples from the batches diagnosed as positively infected were assessed with CS-ELISA, and in parallel with the *Cyt b *based PCR method (hereafter *Cytb*-PCR). Laboratory reared unfed *An. dirus *A mosquitoes were included in both tests as negative control. CS-ELISA and *Cytb*-PCR tests using *P. ovale *and *P. malariae *infected mosquitoes could not be performed due to lack of representative samples. Therefore, for artificially infected mosquitoes, the current study mainly focused on *P. vivax *and *P. falciparum*.

### CS-ELISA

CS-ELISA was performed using the kits specific for *P. vivax*-210 (Pv-210), *P. vivax*-247 (Pv-247) and *P. falciparum *(Centers for Disease Control and Prevention, Atlanta, USA) following manufacturer's instruction with slight modification. Those were: microtitre plates were incubated overnight at 4°C with capture Mab; 37°C for 2 hours after adding test samples, positive and negative controls; and 37°C for 1 hour with peroxidase-linked Mab. Beside these, TMB Microwell Peroxidase Substrate System (KPL, Maryland, USA) was used as substrate solution and ODs were measured using Spectramax 340PC^384 ^(Molecular Devices, CA, USA) at 450 nm. Other conditions were as instructed by the manufacturer. Standard curves were developed to extrapolate the concentrations of test samples by titration of positively controlled antigens of each species.

### DNA extraction from mosquitoes

The whole body of the individual mosquitoes were placed separately in 1.5 ml Eppendorf tubes and grinded with pestles in 50 μl blocking buffer (1% bovine serum albumin, 0.5% casein and 0.002% phenol red made up in 0.01 mol/l Dulbecco's phosphate buffer saline (PBS)) containing 0.5% Nonidet P-40. It is noteworthy that this is the same grinding solution using the BSA/casein blocking buffer as mentioned in the CS-ELISA manufacturer's protocol except that here Nonidet P-40 has been used instead of IGEPAL CA-630. The pestles were rinsed again with 200 μl blocking buffer. An aliquot of 50 μl was used for CS-ELISA. The remaining portion of the homogenate was used to extract genomic DNA for PCR using IsoQuick Nucleic Acid Extraction Kit (ORCA Research Inc., WA, USA) with slight modification of the total DNA extraction protocol provided by the manufacturer. Briefly, instead of using REAGENT A (Sample buffer) 200 μl of REAGENT 1 (Lysis Solution) was directly added to the homogenates and then DNA extraction was continued as mentioned in the protocol. Finally, the product was eluted in 500 μl of TE, incubated in a 100°C water bath for 10 minutes and centrifuged at 15000 rpm for 10 second. An incubation of the extracted product at -20°C for at least 1 hour before PCR amplification was found to be useful for increasing the yield.

### Primer selection

The primers were manually selected for the amplification of a partial *Cyt b *gene, reviewing the representative *Plasmodium *sequences obtained from GenBank using Primaclade [32] and according to the standard guidelines [33]. Primer sequences were further checked for compatibility with the target sequences using web-based software Primer3 v.0.4.0 [34]. Properties of the primers, such as melting temp (*Tm*), GC content and self-complementary patterns were also evaluated to minimize the possibility of self-dimerization or hetero-dimerization using Primer3 and NetPrimer [35]. Finally, primers MitF2 (5'-TGAGTTATTGGGGTGCAACTG-3') and MitR2 (5'-TGTTTGCTTGGGAGCTGTAA-3') showed most potentiality to amplify a suitable 729 bp fragment of the *Cyt b *gene.

### *Cytb*-PCR conditions

The PCR reactions (25 μl) contained 1 μl genomic DNA, 1× PCR buffer (10 mM tris-HCl pH 9.0, 50 mM KCl and 0.1% Triton^® ^X-100), 2.5 mM MgCl_2_, 0.5 μmol of each primer, 200 μmol of each dNTP and 1.25 unit of *Taq *polymerase (Promega, WI, USA). The amplification was performed in a Takara Dice thermal cyler using the temperate cycling conditions of: 4 min at 94°C, followed by 35 cycles of 94°C for 40 seconds, 61°C for 40 seconds and 72°C for 1 min, finally 72°C for 10 min. The amplified DNA was loaded onto 1% or 2% agarose gel with λ hind III marker or 100 bp ladder, respectively and ran for 30 min. The gel was then stained with ethidium bromide for 30 min, and visualized on a UV transilluminator. Since the designed primers were novel, we conducted complete sequence analysis of the PCR product to ensure that the target region has amplified. The products were purified in 5% acrylamide gel following [36] with slight modification. Briefly, excised segments of the gels were grinded with pestle by placing in 1.5 ml Eppendorf tube, then 1 ml gel elution buffer (for directions see [36]) was added and shook overnight at 37°C. The tubes were centrifuged at 15,000 rpm for 5 minutes at 4°C and supernatants were collected. Precipitates were rinsed with 0.5 ml gel elution buffer, centrifuged and supernatants were collected again as before. The supernatants were then purified twice with 100% and 70% ethanol, vacuum dried and suspended in 20 μl TE buffer. The products were cycle-sequenced in both directions with corresponding PCR primers (0.16 μmol) using a BigDye^® ^Terminator v1.1 Cycle Sequencing kit (Applied Biosystems, CA, USA). Sequences were run on an ABI prism™ 377 DNA Sequencer (Applied Biosystems, CA, USA). Forward and reverse strands were assembled for each individual sample in the SEQMANII version 3.6.0 (DNASTAR, Inc.) sequence editor program and a single sequence was defined. Finally, the sequence identity was confirmed by BLAST search in GenBank. In *Cytb*-PCR, DNA extracted from either of the *P. vivax *and *P. falciparum *infected bloods with known DNA concentration were used as a positive control.

### Sensitivity and specificity

To define the lower detection limit of the *Cytb*-PCR, samples with a known number of parasites for each species were serially diluted. That is, templates were prepared by a ten-fold serial dilution using TE buffer, ranged from no dilution (2 ng/μl) to as low as 1:100,000 dilution (20 fg/μl, equivalent to 1 parasite genomes) (e.g. [1,29]). Beside this, to detect the specificity of the method, patients' blood infected with *P. ovale *and *P. malariae *were also used. For specificity, blood collected from healthy donors was also assessed. As mentioned earlier, DNA extracted from laboratory reared unfed *An. dirus *A mosquitoes as well as blanks (no templates) were included in every PCR run to confirm the specificity, and to rule out carry over contaminations.

### Effect of preservation methods

It is noteworthy that all laboratory reared mosquitoes used in this study were killed by freezing and preserved in -80°C immediately for further procedures. However, to detect the effect of preserved materials, ten frozen mosquitoes from each of the batch were preserved in ethanol or on silica gel for 1 month. DNA extraction and PCR detection for *Plasmodium *was performed using these samples in the similar ways as mentioned in the earlier part of this section.

### RFLP designing and conditions

RFLP was designed for the obtained sequences from the samples by web-based software RestrictionMapper [37]. Expected products were designed to differ in size at least 100 bases for unmistakable identification on agarose gel [38], and *Pvu *II was found to be a suitable restriction enzyme for this purpose. Only, the *P. falciparum *sequences possess a single restriction site for *Pvu *II, which should cut the sequences only one time and should generate two bands. The PCR-RFLP was performed using 1 μl of this *Pvu *II (Takara Bio Inc., Japan), 2 μl of 10× M buffer, 5 μl of the PCR product and 12 μl of sterilized distilled water. The reaction mixture was incubated at 37°C for one hour. The digested products were ran in agarose gel (2%), stained and visualised similar to those of the PCR products mentioned above.

### Mosquito collection for field trial

Mosquitoes were collected by human bait method from three areas (Kaboibus, Wingei, and Jawia) of East Sepic Province of Papua New Guinea in February 2002 and February 2003. Specimens were identified morphologically as members of the *An. punctulatus *group [6,39,40] and were preserved in ethanol. These samples were also processed and PCR detection for *Plasmodium *was performed as mentioned in the earlier part of this section. Simultaneously, mosquito species were identified by species-specific PCR-RFLP as described previously [15,41].

### Nested PCR

Diagnostic PCR test for detecting *Plasmodium *from the naturally infected mosquitoes and some selected artificially infected mosquitoes was carried out as described previously [30].

### Statistical analysis of test performance

In this study, CS-ELISA was considered as the 'gold standard' for the artificially infected mosquitoes and *Cytb*-PCR was compared with this CS-ELISA. The agreement between CS-ELISA and *Cytb*-PCR was determined by calculating Cohen's Kappa values with 95% CI in SPSS for windows version 10.0.1 (SPSS Inc. Chicago, IL). The same approach was also followed for the naturally infected mosquitoes. Here, nested PCR method described by Snounou *et al *[30] was considered as the reference method.

## Results

### Comparison between CS-ELISA and *Cytb*-PCR

Mosquitoes were artificially infected with a total of eight isolates of either *P. vivax *(five isolates) or *P. falciparum *(three isolates), were selected to include in this study. Representative mosquitoes from the batches were screened using CS-ELISA. CS-ELISA further differentiated them into three strains as: Pv-210 (two isolates), Pv-247 (three isolates) and *P. falciparum *(three isolates). However, for simplicity of explanation, the findings were coalesced as groups (Pv-210, Pv-247 and *P. falciparum*), rather than as isolates.

Finally, 122 mosquitoes consisting of 90 infected blood-fed (30 mosquitoes each from Pv-210, Pv-247 and *P. falciparum *group) and 32 unfed (16, 8 and 8 mosquitoes, respectively for Pv-210, Pv-247 and *P. falciparum *group) mosquitoes were assessed for their infection rates using CS-ELISA. CS-ELISA for Pv-210 group detected positive infection in all 30 infected blood-fed mosquitoes and no infection in all 16 unfed mosquitoes (Table 1). Similarly, for Pv-247 and *P. falciparum *groups out of 30 infected blood-fed mosquitoes, CS-ELISA showed positive infection in 19 and 18 mosquitoes, respectively. As expected no infection was detected in any of the eight unfed mosquitoes each for Pv-247 and *P. falciparum *(Table 1).

**Table 1:** Results of the comparisons between CS-ELISA (ELISA) and *Cytb*-PCR (PCR) using artificially infected mosquitoes

	Pv-210		Pv-247		Pf	
	ELISA	PCR	ELISA	PCR	ELISA	PCR

Unfed mosquitoes detected negative	16	16	8	8	8	8

Unfed mosquitoes detected positive	0	0	0	0	0	0

Blood fed mosquitoes detected negative	0	3	11	14	12	14

Blood fed mosquitoes detected positive	30	27	19	16	18	16

Pv-210, Pv-247 and Pf are *P. vivax *210, *P. vivax *247 and *P. falciparum*, respectively.

**Figure 1:**
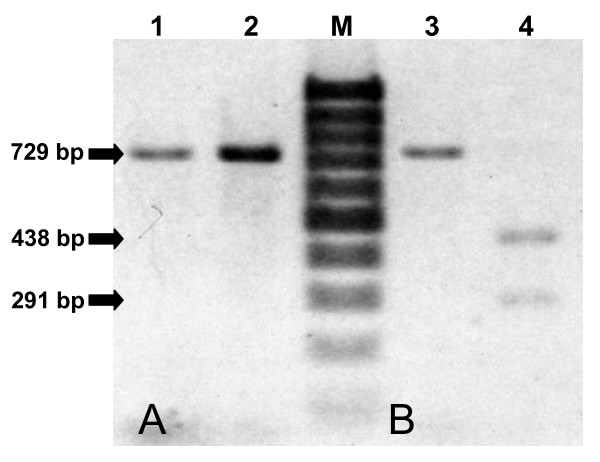
**Banding pattern of the *Cytb*-PCR using artificially infected mosquitoes**. (A) Image showing 729 bp *Cytb*-PCR amplified products of the DNA extracted from the mosquitoes artificially infected with *P. vivax *(lane 1) and *P. falciparum *(lane 2). (B) The same samples after PCR-RFLP showing a single 729 bp fragment specific for *P. vivax *(lane 3), and two 438 bp and 291 bp fragments specific for *P. falciparum *(lane 4). The products were run on 2% agarose gel with 100 bp ladder as molecular size marker (M).

The same samples were further tested to verify the detection ability of the *Cytb*-PCR. The successfully amplified products gave strong bands at 729 basepair (bp) position on a 2% agarose gel and were considered as positive test (Figure 1A), whereas samples, which failed to produce visible bands, were considered as negative. Among the 30, 19 and 18 CS-ELISA-positive mosquitoes from Pv-210, Pv-247 and *P. falciparum *groups, *Cytb*-PCR detected respectively 27, 16 and 16 mosquitoes as positive, and all 32 unfed mosquitoes were detected as negative (Table 1). The comparison revealed a close agreement between the 'gold standard' CS-ELISA and *Cytb*-PCR (κ = 0.862, 0.842 and 0.894, respectively for Pv-210, Pv-247 and *P. falciparum *groups). Nevertheless, the eight CS-ELISA-positive mosquitoes detected negative by *Cytb*-PCR were further evaluated using nested PCR. All of them were again detected negative by this method.

Subsequently, 10 of the *Cytb*-PCR positive products from each group were sequenced further, which resulted in to two different sequences. The BLAST search confirmed their identity as *P. vivax *and *P. falciparum*, and revealed that the *Cytb*-PCR using the MitF2/MitR2 primer set effectively amplified the targeted *Plasmodium Cyt b *gene.

### Sensitivity and specificity

**Figure 2:**
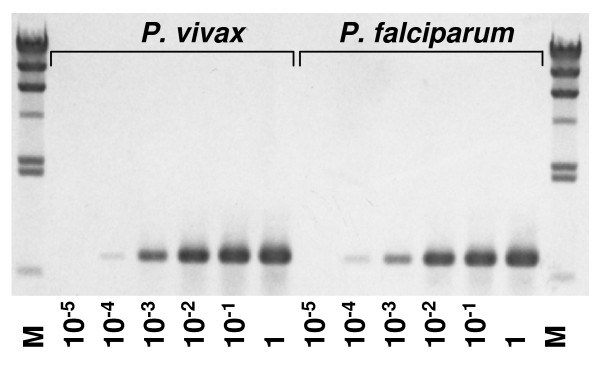
**Sensitivity of the *Cytb*-PCR detection assay**. The serial dilution of DNA from 2 ng/μl (lanes showing 1) to 20 fg/μl (lanes showing 10^-5^) for both *P. vivax *and *P. falciparum *were used as template. Templates for both species containing as low as 1:10,000 dilutions (lanes showing 10^-4^) of DNA, are showing visible bands at 729 bp position. The products were run on 1% agarose gel with λ hind III ladder as molecular size marker (M).

**Figure 3:**
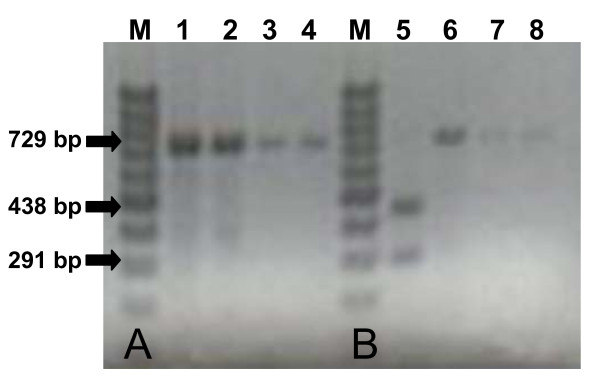
**Banding pattern of the *Cytb*-PCR using infected human blood**. (A) Image showing 729 bp *Cytb*-PCR amplified products of the DNA extracted from human blood infected with *P. falciparum *(lane 1), *P. vivax *(lane 2), *P. malariae *(lane 3) and *P. ovale *(lane 4). (B) The same samples after PCR-RFLP showing a single 729 bp fragment specific for *P. vivax *(lane 6), *P. malariae *(lane 7) and *P. ovale *(lane 8); and two 438 bp and 291 bp fragments specific for *P. falciparum *(lane 5). The products were run on 2% agarose gel with 100 bp ladder as molecular size marker (M).

The analytical sensitivity of the *Cytb*-PCR for both *P. vivax *and *P. falciparum *were at 1:10,000 dilution equivalent to 0.2 pg of DNA that is approximately 10 parasite genomes (Figure 2). The *Cytb*-PCR also successfully amplified the extracted DNAs from *P. ovale *and *P. malariae*, and gave specific bands at position 729 bp on a 2% agarose gel (Figure 3A). No reactions were observed in any of the blood samples from healthy donors, samples of unfed mosquitoes and the blank solutions. These findings confirm that the primers are highly specific for the *Cyt-b *gene of four human *Plasmodium *species, and do not amplify any of the human or mosquito genes.

### Effect of preservation methods

**Figure 4:**
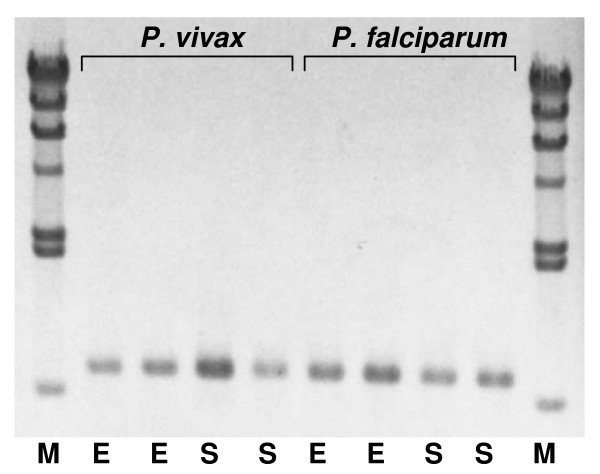
**Banding pattern of the *Cytb*-PCR using mosquitoes preserved in ethanol (E) and on silica gel (S)**. Irrespective of preservative materials, *Cytb*-PCR produces bands at 729 bp position for both *P. vivax *and *P. falciparum*. The products were run on 1% agarose gel with λ hind III ladder as molecular size marker (M).

The efficiency of the extraction-detection method was tested using 20 mosquitoes preserved in ethanol (*n *= 10) or on silica gel (*n *= 10). Test results showed equal efficiencies of amplification for detecting both *P. vivax *and *P. falciparum *irrespective of the preserved materials (Figure 4).

### PCR-RFLP

PCR-RFLP has been attempted using the same templates and allowed to digest them with *Pvu *II. As shown in Figures 1B and 3B, 2% agarose gel clearly discriminate the two *Plasmodium *species present in mosquitoes, and differentiated *P. falciparum *from *P. vivax*, *P. ovale *and *P. malariae *present in human blood; with a single band at position 729 bp for *P. vivax*, *P. ovale *and *P. malariae*, and two bands at positions 438 bp and 291 bp. this pattern is expected, as the restriction site for *Pvu *II was only present one time in *P. falciparum*. The obvious differences shown among the *Plasmodium *species suggests the differentiating efficiency of the PCR-RFLP.

### Field trial

**Figure 5:**
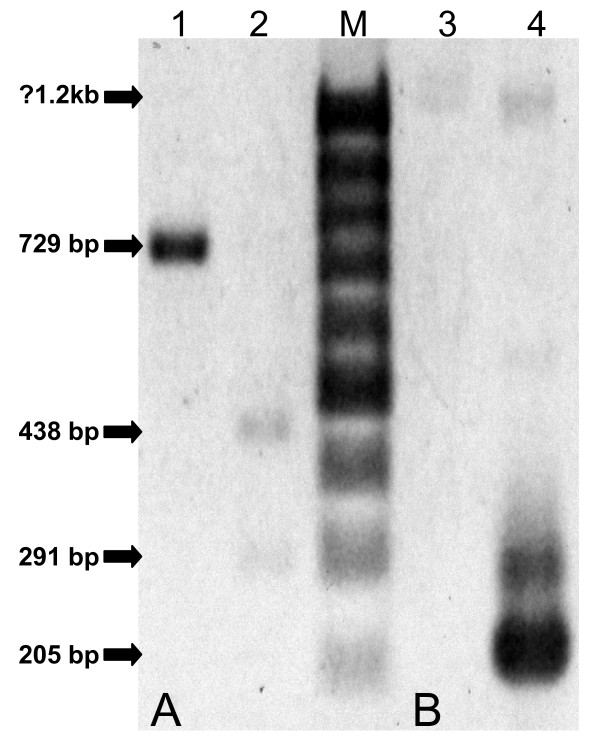
**Detection of *Plasmodium *in field-caught mosquitoes**. (A) *Cytb*-PCR showing a band at position 729 bp (lane 1), and PCR-RFLP showing two bands at positions 438 bp and 291 bp (lane 2) suggesting presence of *P. falciparum *in the sample. (B) Using nested PCR, the same sample after first PCR showing a non-specific band at position 1.2 (lane 3) and after second PCR showing the specific band at position 205 bp (lane 4), concordantly suggesting presence of *P. falciparum *in the sample. The products were run on 2% agarose gel with 100 bp ladder as molecular size marker (M).

A total of 107 mosquitoes were morphologically identified as members of the *An. punctulatus *group and were included in this study. Species specific identification for *An. punctulatus *group using PCR-RFLP, further suggested that *An. punctulatus *made up majority (90.6%, n = 97) of these samples. The remaining was comprised of *An. hinesorum *(4.7%, n = 97), *An. koliensis *(2.8%, n = 3) and *An. farauti *(1.9%, n = 2). *Cytb*-PCR was tested using these samples to detect *Plasmodium*. Only one *An. punctulatus *sample, collected from Kaboibus, gave positive diagnostic band (Figure 5A). Both sequencing and PCR-RFLP confirmed the presence of *P. falciparum *in this sample (Figure 5A). Further screening using nested PCR also detected the same sample as *P. falciparum *positive (Figure 5B). Thus both tests concordantly suggested an overall sporozoite rate of 0.9% (1/107) in the study areas.

## Discussion

The results of this study showed high concordance between CS-ELISA and newly developed *Cytb*-PCR for detecting *P. vivax *and *P. falciparum *sporozoites in artificially infected mosquitoes (κ = 0.862, 0.842 and 0.894, respectively for Pv-210, Pv-247 and *P. falciparum *groups). The lack of agreement between the two assays regarding the eight mosquitoes can be explained by low sporozoite rate in the samples. However, this is unlikely, first, because nested PCR also failed to detect *Plasmodium *from these same samples. Studies have shown that the nested PCR can detect as few as three sporozoites from mosquitoes [16], which is much sensitive than CS-ELISA (50 sporozoites per mosquitoes [22,42]). Second, to compare the sensitivity of this *Cytb*-PCR, the cut-off value of CS-ELISA for this current study was set to a critical limit, 25 sporozoites. This threshold level is also much higher than the lower detection limit of the *Cytb*-PCR, 10 sporozoites per mosquitoes. Finally, to rule out improper DNA extraction, the DNA concentration of the samples was estimated. DNA concentration was found between 10 to 100 ng/μl, which is also within the expected limit. Therefore, although at this point the exact reason is unclear, it could be assumed that these eight samples might be false positive results of the CS-ELISA.

The findings of the field trial were straighter forward than those of the artificially infected mosquitoes. There was complete agreement between the nested PCR and *Cytb*-PCR. Both tests detected the same mosquito as positively infected with *P. falciparum*. This study further suggested that the sporozoite rate is 0.9% in these three areas: Kaboibus, Wingei, and Jawia of East Sepic Province, and that *An. punctulatus *is a vector species. Previous studies conducted in Madang and East Sepic province of Papua New Guinea using CS-ELISA also reported a similar infection rate of *P. falciparum *in 1.1% [13] and 0% – 3.3% [8]*An. punctulatus*. However, these studies also reported both *P. vivax *and *P. falciparum *in *An. punctulatus *including its four other sibling species, *An. farauti*, *An. hinesorum*, *An. farauti *4 and *An. koliensis *[8,13]. In the current study, we could detect neither *P. falciparum *from any of *An. hinesorum*, *An. koliensis *or *An. farauti *nor *P. vivax *from any of the mosquitoes. This might be due to the number of mosquitoes assessed in this study, which were too small to compensate the low sporozoite rate in this region. Further studies using a larger sample size are required to resolve this issue.

PCR based assays for detecting *Plasmodium *from mosquitoes are often considered as a most suitable method where identification and elucidation the genetic variations of parasite species as well as their vector species is required [29]. From this aspect *Cytb*-PCR can be an appropriate method than other PCR based methods. Because, in field surveys, as it is often difficult to rear mosquitoes for long period, they are commonly preserved by freezing or (containing) in ethanol or on silica gel, and processed at different times and places. The *Cytb*-PCR reported here remained unaffected by any of the conventional preserved methods, and consistently gave the diagnostic band (Figure 4), whereas nested PCR can produce non-specific bands in mosquitoes stored in ethanol [1].

Beside this, the intensity of the single step *Cytb*-PCR is as strong as that has shown in nested PCR after the second round of amplification (Figure 5). PCR is much linked to the copy number of the target gene, thus large copy number genes are at vintage positions than those of small number [28]. Therefore, it can be assumed that the high sensitivity of *Cytb*-PCR was to some extend achieved by the five-fold higher copy number of *Plasmodium *mitochondrial DNA (~20 copies per cell; [43]) than that of the conventional *SSUrRNA *gene (four copies per haploid genome [28,44]. Moreover, studies have shown that *Cytb *of *Plasmodium *shows some changes in copy number and electron transport activities during their sexual and asexual stages of development [45,46]. The equal efficiencies of detecting *Plasmodium *from mosquitoes and patients' blood suggests that these changes had not significantly affect the performance of the *Cytb*-PCR. This further making the *Cytb *a suitable marker in the situations where both sexual and asexual stages of parasite development for molecular studies concerning parasites genes involving the strain variation, mutations and drug resistance are required [25,47]. For example, *Cyt b *is associated with the efficiency of chemoprophylaxis of falciparum malaria using one of the widely used drugs, atovaquone-proguanil. The 268-Cys mutation of this gene causes treatment failure [48], and therefore, parallel studies using the *Cyt b *gene in both patients and mosquitoes can be useful for adopting prophylactic regime *a priori *in a particular geographical areas. It is noteworthy that the *P. falciparum Cyt b *gene isolated from the *An. punctulatus *of Kaboibus is a wild type and is not registrant to atovaquone-proguanil.

Nevertheless, the complex pattern of gene expression from *SSUrDNA*, variable selective pressure over the developmental stage and presence of some tandem repeats limits its application in molecular studies, whereas, universally available *Cyt b*, is not under such selective pressure for accumulation of polymorphism and that has made it a valuable tool over *SSUrDNA *for genetic studies [49].

**Figure 6:**
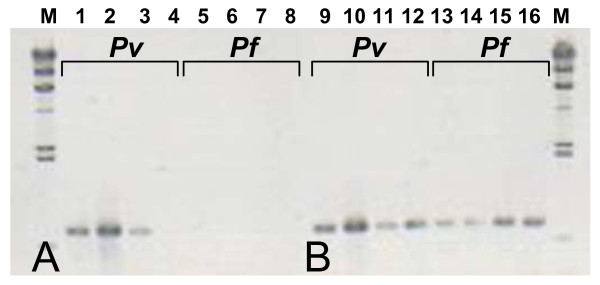
**Differences in amplifications between samples extracted with PBS only and grinding solution containing casein and Nonidet P-40 in PBS**. (A) Image showing some of the *P. vivax *(*Pv*) and *P. falciparum *(*Pf*) samples extracted with PBS only failed to produce representative bands (lanes 4–8). (B) The same batches of mosquitoes extracted with grinding solution containing casein and Nonidet P-40 in PBS showing positive bands (lanes 9–16). The products were run on 1% agarose gel with 100 bp ladder as molecular size marker (M).

PCR assays using the *SSUrRNA *is useful for distinguishing infected from infective mosquitoes [18]. However, this system can underestimate or overlook the true vector in field trials for the mosquitoes, which are collected with their early stage of sporozoite development. To detect this, vectors' DNA extracted from the whole body is required. That is often precluded by a loss of sensitivity to amplify *Plasmodium *using whole mosquitoes due to the presence of some PCR inhibitory materials in the hard exoskeleton of the head and the thorax of mosquitoes [29]. Therefore, removal of the inhibitors either by using only the abdomen or salivary glands, which is impractical for field studies; or by using some reagents to remove the inhibitor is required [29]. Preliminary trials of this study showed that grinding the mosquitoes using only PBS instead of the grinding solution mentioned here, containing casein and Nonidet P-40, often gives false negative results (Figure 6). This observation thus suggests that the additional pre-treating the samples with casein and Nonidet P-40 in PBS effectively eliminated those PCR inhibitors (e.g. [50-52]). It is noteworthy that addition of whole body may paradoxically increase the false positive result (i.e. infected mosquito). However, this extraction method increases the range of detection of a true positive vector.

Despite these advantages, the PCR based methods including this one reported here have some obvious limitations. The first limitation is that PCR is time consuming and requires expensive reagents and equipment, and may not suitable for large-scale epidemiological surveys using large number of mosquitoes only for detecting the presence of *Plasmodium *[47,53]. In such cases CS-ELISA would be the method of choice [8,13], but see also [1,19-21]. This aside, in a conventional way diagnostic methods that require less than one hour are considered as rapid tests [25]. Therefore, PCR based methods are not rapid tests. However, PCR-RFLP can serve the purpose when rapid species detection is required, rather than undertaking the whole process of sequencing (see Methods) or nested PCR. Nevertheless, as the PCR-RFLP reported here could not differentiate between *P. vivax*, *P. ovale *and *P. malariae*, caution should be taken where mixed infection is expected.

Another limitation of the *Cytb*-PCR is that mosquitoes artificially infected only with *P. vivax *and *P. falciparum *were studied here. Although this method successfully amplified the blood samples containing *P. ovale *and *P. malariae*, it is not clear that how efficiently it will amplify these two species from mosquitoes. Conversely, *Cytb*-PCR may non-specifically amplify other *Plasmodium *species in mosquitoes [38], and may give false positive results. Moreover, presence of mutations in the primer binding sites can preclude primer-binding during PCR [54]. Therefore, further studies are required using human blood and mosquito samples infected with other strains of *Plasmodium *species collected from different geographical areas. Lastly, a weakness of PCR is that parasite quantification is not possible [55]. However, the sporozoite rate in mosquitoes is highly variable in the course of time, and rather has less significance on transmitting malaria than the strain of *Plasmodium *([8] and references therein). Therefore, this weakness might not interfere or be of significance for field studies.

In conclusion, this comparative study of the novel *Cytb*-PCR with 'gold standard' CS-ELISA and nested PCR revealed that the result obtained by the *Cytb*-PCR were equivalent to those obtained by the 'gold standards'. Moreover, because of its low detection threshold this assay can be used for the detection and identification even at low parasite levels. Beside this, the PCR-RFLP could clearly distinguish between the *P. vivax *and *P. falciparum*. Therefore, the developed method would be an effective and reliable tool and applicable for detecting the two *Plasmodium *species from the mosquitoes at least collected from Papua New Guinea.

## Competing interests

The authors declare that they have no competing interests.

## Authors' contributions

AUH developed the PCR, designed and performed the study, and drafted the manuscript. SS collected the field-materials, participated in the design and coordination of the study. JS provided *P. falciparum *and *P. vivax *infected mosquitoes obtained from artificial membrane feeding on patients blood. CF performed the PCR-RFLP for mosquito species detection. MA designed the ELISA. MH advised on designing the study and analysis. HO planned the study. All coauthors helped to draft the manuscript and approved the final manuscript.
